# GAP-43 expression correlates with spinal motoneuron regeneration following root avulsion

**DOI:** 10.1186/1749-7221-4-18

**Published:** 2009-10-25

**Authors:** Qiuju Yuan, Bing Hu, Huanxing Su, Kwok-Fai So, Zhixiu Lin, Wutian Wu

**Affiliations:** 1 Department of Anatomy, Li Ka Shing Faculty of Medicine, The University of Hong Kong, Pokfulam, Hong Kong SAR, China; 2 State Key Laboratory of Brain and Cognitive Sciences, The University of Hong Kong, Pokfulam, Hong Kong SAR, China; 3 Research Center of Reproduction, Development and Growth, Li Ka Shing Faculty of Medicine, The University of Hong Kong, Pokfulam, Hong Kong SAR, China; 4 School of Chinese Medicine, Faculty of Science, The Chinese University of Hong Kong, Shatin, N.T, Hong Kong SAR, China; 5 Joint Laboratory for Brain Function and Health (BFAH), Jinan University and The University of Hong Kong, Guangzhou, China

## Abstract

**Background:**

The growth-associated protein GAP-43 plays a crucial role in axonal regeneration in injured neurons.

**Methods:**

We have used immunohistochemistry to investigate the expression of GAP-43 in spinal motoneurons during nerve reconstruction following root avulsion in the neonatal and adult rats.

**Results:**

Following the injury, GAP-43-immunoreactivity (IR) could be found in adult avulsed motoneurons as early as 1 day, increased from 3 to 7 days and reached a maximal level at 2 weeks post-injury. The up-regulation of GAP-43 in adult avulsed motoneurons was accompanied with the axonal regeneration indicated by numerous regenerating motor axons entering the reimplanted ventral root and nerve. In contrast, GAP-43-IR could not be found in the neonatal avulsed motoneurons at any examined post-injury time points. This failure of up-regulation of GAP-43 was coincident with no axonal regeneration in the reimplanted nerve in the neonatal rats.

**Conclusion:**

Close association of GAP-43 expression and capacity of regeneration in reimplanted spinal nerve of avulsed motoneurons suggests that GAP-43 is a potential therapeutic target for treatment of root avulsion of brachial plexus.

## Background

The current treatment for brachial plexus root avulsion is mainly based on nerve transfers and nerve grafts directly implanted into the spinal cord. The results of brachial plexus reconstruction are poor, despite the sophistication of the various methods used [1]. In animals, nerve regeneration into a peripheral nerve (PN) graft after root avulsion was demonstrated in a series of experiments in rats, cats and primates [2-7]. We have previously shown that spinal motoneurons in adult rats can regenerate and reinnervate muscles to recover partial function [8-11]. However, avulsed motoneurons in neonatal rats are unable to regenerate into a PN graft [12], which indicates that intrinsic neuronal factors also determine the regenerative capabilities.

Successfully regenerating neurons in mammalian peripheral nervous system (PNS) undergo a variety of changes in gene expression, for example, the prominent upregulation of growth-associated proteins [13,14]. This regeneration-associated gene (RAG) expression is believed to enhance the growth potential of injured neurons. Sensory neurons exhibit little regeneration of their central axon into a peripheral nerve transplant unless their peripheral axon is also axotomized [15], correlating with the stimulation of RAG expression, such as GAP-43 after axotomy of the peripheral but not of the central axon [16]. In central nervous system, brain-derived neurotrophic factor (BDNF) but not neurotrophin-3 (NT-3) was found to increase the number of axotomized rubrospinal tract neurons that regenerated into grafts of sciatic nerve implanted into the spinal cord at the level of spinal transaction, also correlating with the stimulation of GAP-43 expression after application of BDNF but not of NT-3. Expression of GAP-43 has also been investigated in spinal motoneurons following axonal injury [17]. However, the correlation between GAP-43 expression and regenerative capacity of injured motoneurons has not been well established. The present experiment was designed to study the expression of GAP-43 following unilateral avulsion and implantation of cervical 7 (C7) of brachial plexus in neonatal and adult rats. The potential role of such expression for axonal regeneration of avulsed motoneurons after root avulsion was discussed.

## Materials and methods

Female Sprague-Dawley postnatal day 1 (PN1), and adult rats (220-250 g) were used. Animals were anesthetized under deep hypothermia (for PN1) or with ketamine (80 mg/kg) and xylazine (8 mg/kg) (for adult rats). All surgical interventions and subsequent care and treatment were approved by the Committee on the Use of Live Animals for Teaching and Research of the University of Hong Kong.

Anesthetized animals were placed on the surgical table and a dorsal laminectomy was carried out. The dura was opened and the ventral root and dorsal root with the ganglion of C7 were selectively avulsed from the spinal cord by traction under a surgical microscope following procedures described previously [18]. The site was checked visually to confirm complete avulsion.

For animals received PN reimplantation, the avulsed ventral root was reimplanted following the procedure described in a previous study [10]. Briefly, after avulsion and dorsal root ganglion removing, the ventral root was carefully reimplanted into the ventrolateral aspect of spinal segment C7 with a fine glass probe. Care was taken not to injure the spinal white matter. The dura was closed. The muscles, subcutaneous tissues and skin were closed in separate layers. Following the operation, the animals were allowed to survive for 1, 3, 7, 14 and 28 days, with five rats in each postoperative time period.

At the end of the postoperative survival period, the rats were deeply anesthetized with a lethal dose of ketamine (160 mg/kg) and xylazine (16 mg/kg) and were perfused intracardially with normal saline, followed by 4% paraformaldehyde in 0.1 M phosphate-buffered (PB) (pH 7.4). A 5 mm segment of C7 spinal nerve was dissected before its first branch. The C7 spinal segments were carefully dissected under a dissection microscope in order to avoid damage the implantation area. Tissues were immersion-fixed in the same fixative for 6 h. They were then placed into 30% sucrose in 0.1 M PB overnight. Transverse serial sections of spinal cord at 40 μm were cut and collected in wells containing 0.1 M PB.

The sections were incubated overnight at room temperature with a rabbit polyclonal antibody against GAP-43 (1:500, Chemicon International, Temecula, Calif). After rinsing with PB, they were incubated for 2 hours at room temperature with a goat-anti-rabbit secondary antibody conjugated with Alexa-488 (1:400, Molecular Probes, Eugene, USA). The primary and secondary antibodies were diluted in PBS containing 1% normal goat serum and 0.2% Triton X-100.

After reaction, the sections were mounted on gelatin-coated glass slides and coverslipped in mounting medium (Dako, Denmark). Fluorescent images were captured with Zeiss microscope (Zeiss, Gottingen, Germany) equipped with Spot digital camera (Diagnostic Instruments, Sterling Heights, MI, USA). Numbers of GAP-43-IR motoneurons in every alternate section were counted. All results are expressed as mean ± SD.

Sections immunostained with antibody against GAP-43 were counterstained with neutral red. The number of surviving motoneurons was counted on both the intact and the lesioned sides as described previously [19]. The total number of surviving motoneurons on the lesioned side was expressed as a percentage of the number of motoneurons on the contralateral side.

## Results

### Age-dependent GAP-43-IR expression in avulsed motoneurons

**Figure 1:**
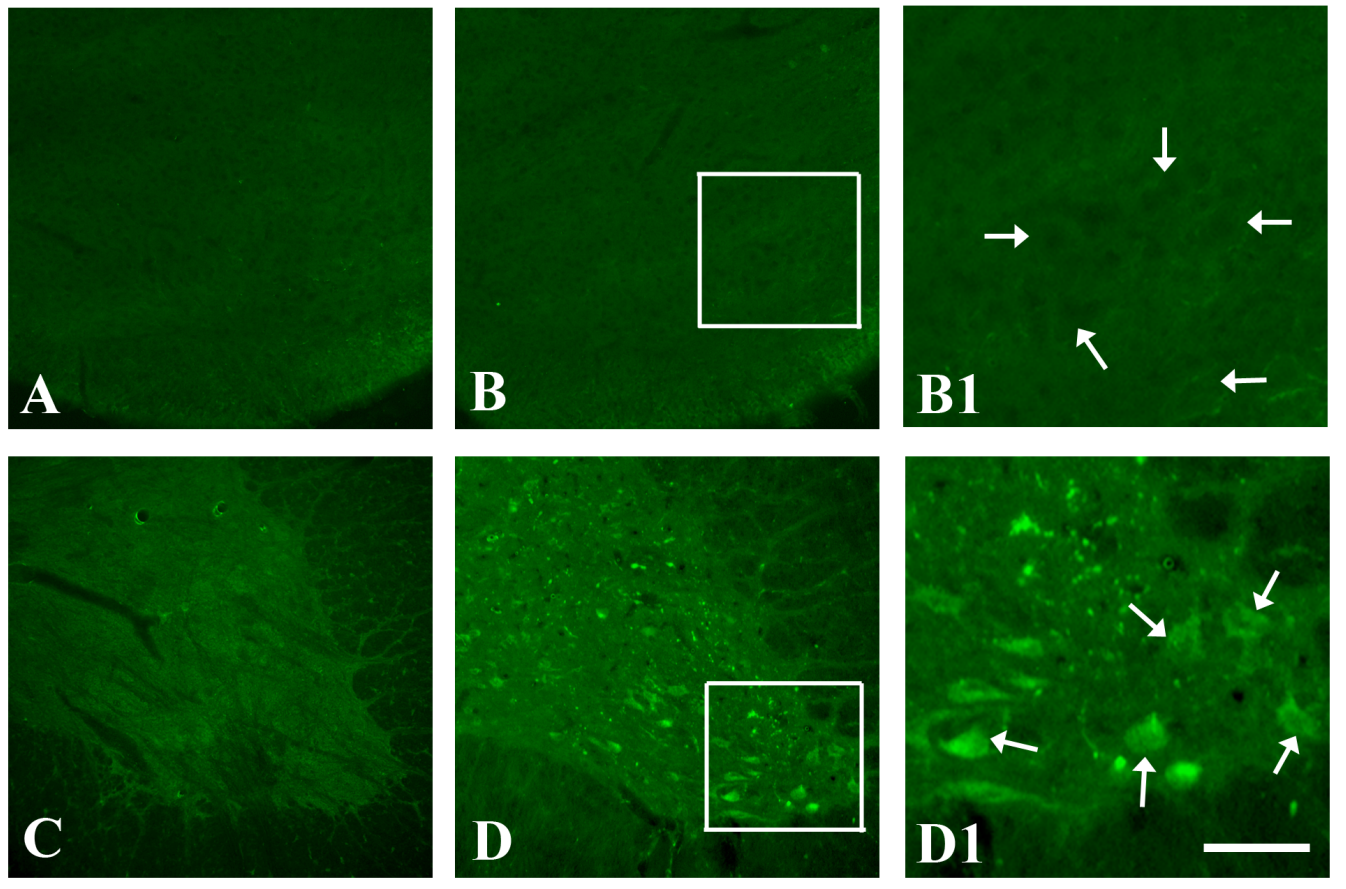
**Representative photomicrographs showing the expression GAP-43 in avulsed motoneurons in the neonatal and adult rats**. No GAP-43-IR was detected in ventral horn of the lesion side in neonatal at 3 days post-injury (B), which was comparable to the age-matched normal control (A). B1 is the enlargement of the square area in the ventral horn of image B showing negative GAP-43-IR of motoneurons (arrows). In contrast, GAP-43-IR was induced in many avulsed motoneurons at 14 days post-injury in the adult animals (D) compared with the adult normal control (C). D1 is the enlargement of the square area in the ventral horn of image D showing positive GAP-43-IR of motoneurons (arrows). Scale bar = 400 μm in A-D, 100 μm in B1 and D1.

No GAP-43-IR motoneurons could be found in normal neonatal or adult rats (Fig 1A, C respectively). Following root avulsion in neonatal animals, GAP-43-IR motoneurons could not be seen in lesion side of ventral horn at all examined post-injury time points following avulsion (Table 1, Fig 1B). In this age of animals, avulsion induced marked motoneuron death within 1 week post-injury (Table 2).

**Table 1:** GAP-43-IR motoneurons in neonatal and adult rats after avulsion.

Survival day(s)	neonatal	adult
1	--	+/--

3	--	+

7	--	+

14	--	++

28	--	--

The staining induction (--, absent; +, moderate; ++, intense) was assessed as compared to the non-operated side using criteria as (--) no GAP-43-IR motoneuron, (+/--) 1-30 GAP-43-IR motoneurons, (+) 31-150 GAP-43-IR motoneurons and (++) > 150 GAP-43-IR motoneurons.

**Table 2:** Survival of motoneurons after root avulsion in neonatal and adult rats.

Day(s) after avulsion	neonatal	adult
1	99.3 ± 5.9	98.1 ± 7.5

3	63.4 ± 4.7	99 ± 4.9

7	5.2 ± 0.9	102.2 ± 6.1

14		89.2 ± 5.7

28		46.4 ± 4.1

Data are expressed as a percentage (mean ± SEM) of the number of motoneurons on the contralateral side, which represent 100%.

In contrast, following spinal root avulsion in adult animals, GAP-43-IR motoneurons in the avulsed ventral horn were present at 1 day post-injury, subsequently increased from 3 to 7 days and peaked at 14 days post-injury (Table 1, Fig 1D). Expression of GAP-43 decreased at 4 week post-avulsion (Table 1). In adult rats, avulsion did not lead to significant motoneuron death until 2 weeks post-injury (Table 2).

### Age-dependent motor axon regeneration following reimplantation of avulsed roots

**Figure 2:**
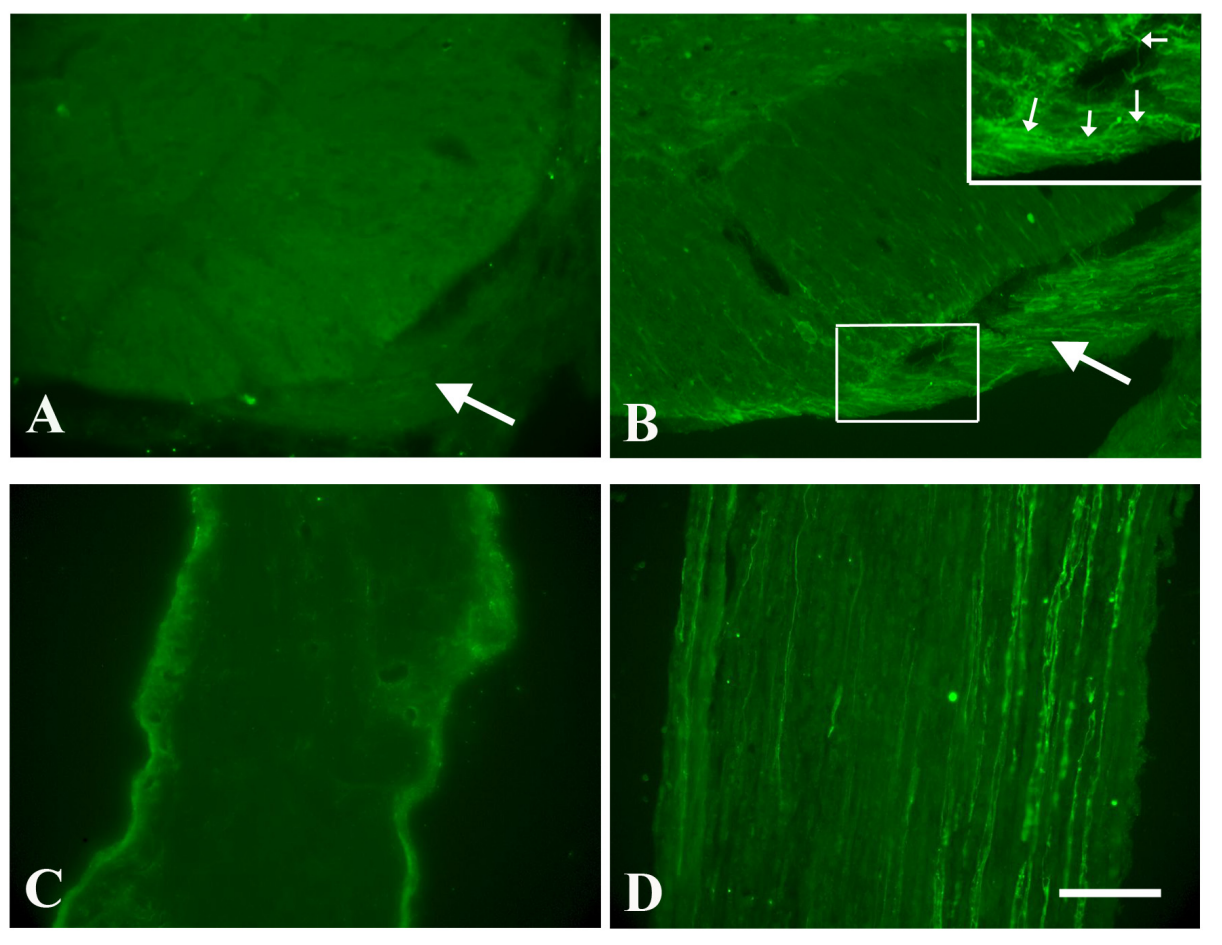
**Representative photomicrographs showing regenerative axons at reimplantation area and root at 3 days (large arrows in A and B) and the C7 spinal nerve at 2 weeks (C, D) post-injury in the neonatal (A, C) and adult (B, D)**. No GAP-43-IR regenerative axons were found in the reimplanted area (A) and the C7 spinal nerve (C) following root avulsion and reimplantation in the neonatal. Numerous GAP-43-IR regenerative motor axons were found in the reimplanted area (B) and the C7 spinal nerve (D) following root avulsion and reimplantation in the adult. Insertion in B is the enlargement of the rectangle area in B showing GAP-43 positive fibers grow into the re-implanted root (small arrows). Scale bar = 100 μm.

To assess whether there is also an age-dependent motor axon regeneration, fiber growth into the implanted ventral roots was investigated. As shown in Fig 2A and 2B (arrow), reimplanted ventral roots contact well with the ventral root exit zone 3 days following reimplantation in the neonatal and adult. No GAP-43-IR fibers were seen in ventral root exit zone and implanted ventral roots in the neonatal rats (Fig 2A). In contrast, numerous GAP-43-IR fibers were found towards and into the reimplanted ventral root from the ventral root exit zone in the adult animals (Fig 2B). At 2 weeks post-implantation, no regenerating axons revealed by GAP-43 immunostaining were observed in reimplanted C7 spinal nerve in the neonatal (Fig 2C). In contrast, many GAP-43-IR axons were found in the adult (Fig 2D).

## Discussion

This study showed that 1) adult but not neonatal motoneurons expressed GAP-43 following root avulsion, 2) GAP-43 was transiently expressed in adult avulsed motoneurons, 3) adult but not neonatal motoneurons could regenerate their avulsed axons into the reimplanted peripheral nerve.

### Age-dependent upregulation of GAP-43 in avulsed motoneurons

It has previously been reported that regenerative capacity for avulsed motoneurons is age-dependent [12]. For example, neonatal motoneurons are unable to regenerate their axons into the transplanted PN graft following root avulsion [12] whereas in adult animals motoneurons are able to regenerate axons into the PN graft [8,10]. In this study, we used root avulsion and reimplantation model and found that adult but not neonatal motoneurons could regenerate their axons into the reimplanted ventral root and spinal nerve. This result further confirms that regenerative capacity for avulsed motoneurons is age-dependent. The poor regeneration in the neonatal rats following root avulsion is in contrast with the situation observed in human. Previous clinical observations have showed that a better functional recovery from the brachial plexus injury at birth compared with that in the adult [20]. However, the extrapolation of experimental data to human situation will have to confront the issue of age comparison between humans and the animals. Although there is no simple answer to making age comparisons between humans and the animals used in animal models [21], Romijn et al [22] uses a variety of measurements and determines that the nervous system of a newborn human is developmentally most comparable to that of a PN13 rat pup. If so, the result observed in a newborn human would be consistent with that in PN13 rat pup. In fact, previous studies have shown that avulsed motoneurons in around PN13 rats can regrow their axons into PN graft [12]. Whether the difference in age-dependent motoneuron regenerative capacity between rats and human is due to different mature stages of rats and human beings needs further investigation.

Successful regeneration depends on upregulation of some molecules [23,24]. Identification of molecules involved in regenerative processes is a key step toward development of therapeutic tools in order to promote functional recovery.

Although many molecules appear to correlate with the neuron's regenerative competence, the most prominent molecular involved in regeneration is GAP-43 [14]. GAP-43 is extensively investigated in CNS and PNS following axonal injury, however, GAP-43 expression in avulsed spinal motoneurons, which are destined to die ultimately, is not investigated.

In this study, we have found that expression of GAP-43 was upregulated in spinal motoneurons and such expression is age-dependent. No GAP-43 expression could be found in neonatal motoneurons following root avulsion. The coincident expression of GAP-43 with robust axonal regeneration in adult and the absence of GAP-43 expression and axonal regeneration in neonatal suggest that GAP-43 plays an important role in regeneration of avulsed spinal motoneurons. The failure of GAP-43 expression in neonatal avulsed motoneurons may be due to the fact that a more rapid motoneuron loss occurs in neonatal rats compared with that in adult rat following root avulsion. However, the fact that GAP-43 was induced in the avulsed spinal motoneurons in adult rats 1 day onward after avulsion implies that 1 day may be a sufficient time interval for a GAP-43 induction. After avulsion at neonatal, although most motoneurons still survived for 1 day after injury, no GAP-43-positive motoneuron was observed. This may exclude the possibility that there was not sufficient time to allow GAP-43 to become manifest in avulsed motoneurons in neonatal rats.

Age-dependent GAP-43 expression in avulsed motoneurons may result from age-dependent expression of calcitonin gene related peptide, which is responsible for encoding growth-associated protein following nerve injury [17]. Calcitonin gene related peptide is upregulated in adult motoneurons after injury, whereas it is downregulated following the same injury in developing animals [17].

### Transient expression of GAP-43 in adult animals

Unlike nerve crush, which preserves the endoneural tube and the continuity of basal lamina, providing neurotrophic support and a physical guide for the proximal axonal ends [25,26], avulsion injury separates motoneurons from all peripheral axons and associated glia. Clinically, it was noted that patients with PN graft transplantation early after the injury had a better outcome than later [7]. Thus, an optimal timing for surgery is an important factor for optimal functional recovery after root avulsion injury. Based on the role of GAP-43 in axonal regeneration, a better understanding of time course of GAP-43 expression in avulsed motoneurons may be essential to develop an optimal time window for surgery repair in order to accelerate the re-connection of the axons with their targets. In the present study, we found that GAP-43 was transiently expressed in adult rats following root avulsion within two weeks and returned to minimal level four weeks post-injury. Therefore, we suggest that optimal timing for surgery repair is around 2 weeks post-injury. Delayed implantation of a PN graft up to 3 weeks post-injury does not significantly affect regeneration even if motoneuron survival is reduced at those surgery time points following spinal root avulsion in adult rats [11,27]. Delayed implantation of a PN graft at 4 weeks post-injury results in a poor regeneration of avulsed motoneurons (data not shown). The fact that avulsed spinal motoneurons have duration for retaining the ability to regenerate may be due to transient expression of GAP-43 of avulsed motoneurons.

## Conclusion

Close association of GAP-43 expression and capacity of regeneration in reimplanted spinal nerve of avulsed motoneurons suggests that GAP-43 is a potential therapeutic target for treatment of root avulsion of brachial plexus.

## Abbreviations

IR: Immunoreactivity; PN: peripheral nerve; PNS: peripheral nervous system; RAG: regeneration-associated gene; BDNF: brain-derived neurotrophic factor; NT-3: neurotrophin-3; PB: phosphate-buffered.

## Competing interests

The authors declare that they have no competing interests.

## Authors' contributions

QY performed experiments, collected and analyzed data, was involved in study design and wrote the manuscript; BH. collected and analyzed data; HS. collected and analyzed data; KFS analyzed data; ZL analyzed data; WW designed the study, collected and analyzed data, wrote the manuscript. All authors read and approved the final manuscript.
